# Supplementary material for: Educational attainment and chronic HIV treatment adherence in southern and eastern Africa
Source: PLoS One. 2026 May 14;21(5):e0348947. doi: 10.1371/journal.pone.0348947 (PMC13175329; doi:10.1371/journal.pone.0348947)
Supplement: S1 Text — (DOCX) [file pone.0348947.s001.docx]

**Supporting Information. Sensitivity Analyses**

**Table 1. Sensitivity Analysis: Associations between education *levels* and viral load suppression (viral load suppression) among people living with HIV who initiated antiretroviral therapy, adjusted odds ratios (AOR) with 95% confidence intervals (CI)**

|  | **Revised Model 2: With other socioeconomic status**  **(CI)** |
| --- | --- |
| **Education level** |  |
| No Schooling | (ref) |
| Some Primary | 1.216 |
|  | (0.886,1.667) |
| Completed Primary | 1.044 |
|  | (0.751,1.451) |
| Some Secondary | 1.180 |
|  | (0.859,1.620) |
| Completed Secondary | 1.275 |
|  | (0.796,2.041) |
| Tertiary | 0.997 |
|  | (0.603,1.648) |
| **Gender** |  |
| Male | (ref) |
| Female | 1.608^***^ |
|  | (1.340,1.930) |
|  |  |
| **Age (years, centered at 15)** | 1.031^***^ |
|  | (1.022,1.040) |
|  |  |
| Never married/partnered | (ref) |
| Previously married/partnered | 1.142 |
|  | (0.871,1.499) |
| Currently married/partnered | 0.987 |
|  | (0.727,1.341) |
|  |  |
| **Current schooling** |  |
| Out of school | (ref) |
| In school | 1.066 |
|  | (0.751,1.514) |
|  |  |
| **Children in home (#)** | 1.020 |
|  | (0.981,1.061) |
| **Rurality** |  |
| Urban home |  |
| Rural home | 1.114 |
|  | (0.895,1.387) |
|  |  |
| **Employed in the past year** |  |
| Not employed | (ref) |
| Employed | 1.147 |
|  | (0.959,1.373) |
|  |  |
| **Wealth quintile** |  |
| Lowest wealth | (ref) |
| Low wealth | 1.277^^^ |
|  | (0.969,1.683) |
| Medium wealth | 1.263^^^ |
|  | (0.964,1.655) |
| High wealth | 1.250 |
|  | (0.942,1.659) |
| Highest wealth | 1.375^^^ |
|  | (0.972,1.945) |
|  |  |
| **Country** |  |
| Lesotho | (ref) |
| Malawi | 1.268 |
|  | (0.945,1.700) |
| Namibia | 1.426^*^ |
|  | (1.088,1.869) |
| Tanzania | 0.946 |
|  | (0.710,1.260) |
| Uganda | 0.662^**^ |
|  | (0.526,0.834) |
| Zambia | 1.014 |
|  | (0.797,1.290) |
| Zimbabwe | 0.765^*^ |
|  | (0.619,0.945) |
|  |  |
| *N* | 12198 |

**Table 2. Sensitivity Analysis: Multivariable associations between years of education and viral load suppression (viral load suppression) among people living with HIV who initiated antiretroviral therapy, adjusted odds ratios (AOR) with 95% confidence intervals (CI), by country**

|  | **Lesotho** | **Malawi** | **Namibia** | **Tanzania** | **Uganda** | **Zambia** | **Zimbabwe** |
| --- | --- | --- | --- | --- | --- | --- | --- |
| **Education (years)** | 1.031 | 1.033 | 1.013 | 1.003 | 0.985 | 1.021 | 1.004 |
|  | (0.987,1.076) | (0.967,1.104) | (0.970,1.057) | (0.931,1.081) | (0.942,1.029) | (0.955,1.091) | (0.954,1.056) |
| **Gender** |  |  |  |  |  |  |  |
| Male (ref) | |  |  |  |  |  |  |
| Female | 1.106 | 1.28 | 1.383 | 2.202* | 1.643* | 1.543* | 1.627** |
|  | (0.787,1.555) | (0.777,2.108) | (0.924,2.069) | (1.138,4.261) | (1.119,2.413) | (1.007,2.363) | (1.241,2.132) |
|  |  |  |  |  |  |  |  |
| **Age (years, centered at 15)** | 1.050*** | 1.019 | 1.035*** | 1.017 | 1.032*** | 1.059*** | 1.039*** |
|  | (1.034,1.066) | (0.996,1.042) | (1.018,1.052) | (0.991,1.043) | (1.014,1.049) | (1.037,1.082) | (1.025,1.054) |
| **Relationship status** | | |  |  |  |  |  |
| Never married/partnered (ref) | | | |  |  |  |  |
| Previously married/partnered | 1.036 | 1.184 | 0.962 | 1.555 | 0.969 | 1.588^ | 0.802 |
|  | (0.703,1.527) | (0.524,2.678) | (0.665,1.392) | (0.704,3.435) | (0.471,1.993) | (0.952,2.649) | (0.447,1.438) |
| Currently married/partnered | 0.874 | 1.739 | 0.615^ | 1.056 | 0.757 | 1.151 | 0.787 |
|  | (0.569,1.343) | (0.763,3.965) | (0.359,1.051) | (0.423,2.636) | (0.353,1.623) | (0.617,2.149) | (0.429,1.445) |
| **Current school status** | | |  |  |  |  |  |
| Not in school (re) | |  |  |  |  |  |  |
| In school | 1.047 | 0.773 | 0.809 | 1.021 | 0.922 | 1.271 | 1.356 |
|  | (0.686,1.597) | (0.310,1.927) | (0.419,1.561) | (0.360,2.893) | (0.375,2.267) | (0.609,2.651) | (0.571,3.221) |
|  |  |  |  |  |  |  |  |
| **Children in home (#)** | 0.964 | 0.987 | 1.05 | 1.008 | 1.068 | 1.021 | 0.979 |
|  | (0.872,1.065) | (0.865,1.126) | (0.976,1.130) | (0.905,1.123) | (0.984,1.158) | (0.942,1.106) | (0.912,1.052) |
| **Rurality** |  |  |  |  |  |  |  |
| Urban home (ref) | |  |  |  |  |  |  |
| Rural home | 1.328 | 1.319 | 0.956 | 0.951 | 1.136 | 1.809* | 1.061 |
|  | (0.914,1.929) | (0.684,2.543) | (0.592,1.544) | (0.561,1.613) | (0.682,1.893) | (1.038,3.152) | (0.577,1.950) |
| **Employment in past year** | | |  |  |  |  |  |
| Not employed (ref) | | |  |  |  |  |  |
| Employed | 0.859 | 0.928 | 0.821 | 1.525^ | 1.394^ | 0.781 | 1.09 |
|  | (0.635,1.163) | (0.536,1.608) | (0.565,1.195) | (0.923,2.520) | (0.945,2.058) | (0.528,1.155) | (0.810,1.467) |
| **Wealth quintile** | |  |  |  |  |  |  |
| Lowest wealth (ref) | | |  |  |  |  |  |
| Low wealth | 1.219 | 1.012 | 0.979 | 1.833 | 1.239 | 1.448 | 1.219 |
|  | (0.787,1.888) | (0.395,2.590) | (0.580,1.654) | (0.677,4.964) | (0.704,2.182) | (0.694,3.022) | (0.831,1.788) |
| Medium wealth | 1.252 | 0.784 | 1.05 | 1.049 | 2.170** | 3.426** | 0.972 |
|  | (0.765,2.050) | (0.322,1.906) | (0.560,1.966) | (0.422,2.606) | (1.262,3.733) | (1.615,7.271) | (0.655,1.443) |
| High wealth | 1.396 | 0.779 | 1.886 | 1.264 | 1.697* | 2.720* | 1.038 |
|  | (0.853,2.284) | (0.300,2.019) | (0.818,4.346) | (0.503,3.177) | (1.026,2.806) | (1.234,5.996) | (0.571,1.885) |
| Highest wealth | 1.394 | 1.028 | 0.428^ | 0.904 | 2.105* | 3.536** | 1.228 |
|  | (0.756,2.568) | (0.363,2.906) | (0.174,1.051) | (0.302,2.708) | (1.056,4.196) | (1.533,8.156) | (0.638,2.365) |
| N | 2628 | 1721 | 2127 | 1124 | 1301 | 1631 | 2714 |
| 95% confidence intervals in brackets | | | | | | |  |

^ p < 0.10, * p < 0.05, ** p < 0.01, *** p < 0.001

**Table 3. Sensitivity Analysis: Multivariable associations with viral load suppression with gender moderation, model 2, adjusted odds ratios (AOR) with 95% confidence intervals (CI), by country**

|  | **Lesotho** | **Malawi** | **Namibia** | **Tanzania** | **Uganda** | | **Zambia** | | **Zimbabwe** | |
| --- | --- | --- | --- | --- | --- | --- | --- | --- | --- | --- |
| **Education (years)** | 1.026 | 1.028 | 1.024 | 0.993 | 0.965 | | 1.036 | | 1.032 | |
|  | (0.967,1.089) | (0.936,1.129) | (0.947,1.107) | (0.892,1.105) | (0.906,1.028) | | (0.924,1.163) | | (0.943,1.128) | |
| **Gender** |  |  |  |  |  | |  | |  | |
| Male (ref) |  |  |  |  |  | |  | |  | |
| Female | 1.031 | 1.222 | 1.574 | 1.998 | 1.322 | | 1.874 | | 2.555* | |
|  | (0.596,1.782) | (0.557,2.677) | (0.753,3.290) | (0.715,5.584) | (0.696,2.513) | | (0.683,5.143) | | (1.042,6.268) | |
| **Female x Education** | 1.01 | 1.008 | 0.982 | 1.017 | 1.034 | | 0.977 | | 0.95 | |
|  | (0.939,1.087) | (0.912,1.114) | (0.892,1.080) | (0.882,1.174) | (0.958,1.117) | | (0.864,1.104) | | (0.857,1.052) | |
|  |  |  |  |  |  | |  | |  | |
| **Age (years, centered at 15)** | 1.050*** | 1.019^ | 1.035*** | 1.017 | 1.032*** | | 1.059*** | | 1.039*** | |
|  | (1.034,1.066) | (0.996,1.042) | (1.018,1.052) | (0.992,1.044) | (1.015,1.050) | | (1.037,1.082) | | (1.025,1.053) | |
| **Relationship status** | |  |  |  |  | |  | |  | |
| Never married/partnered (ref) | | |  |  |  | |  | |  | |
| Previously married/partnered | 1.036 | 1.185 | 0.96 | 1.561 | 0.986 | | 1.589^ | | 0.801 | |
|  | (0.702,1.528) | (0.524,2.680) | (0.664,1.387) | (0.704,3.462) | (0.480,2.028) | | (0.951,2.654) | | (0.447,1.433) | |
| Currently married/partnered | 0.874 | 1.74 | 0.614^ | 1.057 | 0.77 | | 1.145 | | 0.783 | |
|  | (0.569,1.343) | (0.763,3.968) | (0.359,1.049) | (0.423,2.641) | (0.359,1.649) | | (0.614,2.135) | | (0.429,1.431) | |
| **Current school status** | |  |  |  |  | |  | |  | |
| Not in school (ref) | |  |  |  |  | |  | |  | |
| In school | 1.048 | 0.776 | 0.81 | 1.017 | 0.909 | | 1.267 | | 1.382 | |
|  | (0.688,1.598) | (0.311,1.935) | (0.421,1.558) | (0.359,2.886) | (0.370,2.232) | | (0.606,2.647) | | (0.582,3.282) | |
| **Children in home (#)** | 0.964 | 0.987 | 1.05 | 1.009 | 1.068 | | 1.021 | | 0.979 | |
|  | (0.872,1.066) | (0.865,1.126) | (0.976,1.130) | (0.905,1.124) | (0.984,1.159) | | (0.942,1.106) | | (0.911,1.052) | |
| **Rurality** |  |  |  |  |  | |  | |  | |
| Urban home (ref) | |  |  |  |  | |  | |  | |
| Rural home | 1.325 | 1.317 | 0.954 | 0.947 | 1.13 | | 1.804* | | 1.066 | |
|  | (0.911,1.928) | (0.685,2.533) | (0.590,1.541) | (0.558,1.606) | (0.678,1.882) | | (1.034,3.150) | | (0.577,1.970) | |
| **Employment in past year** | |  |  |  |  | |  | |  | |
| Not employed (ref) | |  |  |  |  | |  | |  | |
| Employed | 0.859 | 0.928 | 0.824 | 1.525^ | 1.384^ | | 0.782 | | 1.096 | |
|  | (0.635,1.162) | (0.535,1.609) | (0.567,1.197) | (0.924,2.518) | (0.939,2.040) | | (0.528,1.156) | | (0.814,1.475) | |
| **Wealth quintile** | |  |  |  |  | |  | |  | |
| Lowest wealth (ref) | |  |  |  |  | |  | |  | |
| Low wealth | 1.22 | 1.01 | 0.982 | 1.827 | 1.23 | | 1.453 | | 1.232 | |
|  | (0.788,1.889) | (0.394,2.589) | (0.581,1.661) | (0.679,4.917) | (0.698,2.165) | | (0.698,3.023) | | (0.839,1.808) | |
| Medium wealth | 1.252 | 0.782 | 1.052 | 1.045 | 2.168** | | 3.441** | | 0.974 | |
|  | (0.765,2.050) | (0.322,1.897) | (0.562,1.971) | (0.425,2.574) | (1.260,3.731) | | (1.622,7.298) | | (0.655,1.447) | |
| High wealth | 1.396 | 0.777 | 1.887 | 1.26 | 1.684* | | 2.717* | | 1.049 | |
|  | (0.853,2.285) | (0.300,2.009) | (0.820,4.345) | (0.506,3.138) | (1.017,2.786) | | (1.232,5.995) | | (0.574,1.916) | |
| Highest wealth | 1.39 | 1.022 | 0.427^ | 0.901 | 2.091* | | 3.533** | | 1.26 | |
|  | (0.753,2.566) | (0.365,2.863) | (0.174,1.050) | (0.303,2.681) | (1.047,4.178) | | (1.532,8.148) | | (0.652,2.435) | |
| N | 2454 | 1584 | 2070 | 1007 | 1195 | | 1433 | | 2455 | |
| 95% confidence intervals in brackets | | | | | | | | | |  |
| ^ p < 0.10, * p < 0.05, ** p < 0.01, *** p < 0.001 | | | | | |  | |  | |  |

**Table 4. Sensitivity Analysis: Multivariable associations with viral load suppression with age moderation, model 2, adjusted odds ratios (AOR) with 95% confidence intervals (CI), by country**

|  | **Lesotho** | **Malawi** | **Namibia** | **Tanzania** | **Uganda** | **Zambia** | **Zimbabwe** |
| --- | --- | --- | --- | --- | --- | --- | --- |
| **Education (years)** | 0.998 | 0.98 | 1.06 | 0.911 | 0.981 | 0.974 | 0.948 |
|  | (0.902,1.104) | (0.838,1.145) | (0.946,1.188) | (0.769,1.080) | (0.891,1.080) | (0.864,1.097) | (0.850,1.056) |
| **Gender** |  |  |  |  |  |  |  |
| Male (ref) |  |  |  |  |  |  |  |
| Female | 1.108 | 1.302 | 1.369 | 2.349* | 1.645* | 1.560* | 1.652*** |
|  | (0.787,1.560) | (0.790,2.146) | (0.916,2.046) | (1.216,4.540) | (1.118,2.421) | (1.020,2.388) | (1.261,2.163) |
| **Age (years, centered at 15)** | 1.039* | 1.007 | 1.048** | 0.996 | 1.031* | 1.042* | 1.021 |
|  | (1.005,1.074) | (0.966,1.050) | (1.014,1.083) | (0.951,1.044) | (1.002,1.060) | (1.001,1.084) | (0.989,1.054) |
| **Age (years, centered at 15) x Education (years)** | 1.001 | 1.002 | 0.998 | 1.004 | 1.000 | 1.002 | 1.002 |
|  | (0.998,1.005) | (0.996,1.008) | (0.994,1.002) | (0.997,1.011) | (0.996,1.004) | (0.997,1.007) | (0.998,1.006) |
| **Relationship status** | |  |  |  |  |  |  |
| Never married/partnered (ref) | | |  |  |  |  |  |
| Previously married/partnered | 1.024 | 1.138 | 0.965 | 1.458 | 0.967 | 1.529^ | 0.763 |
|  | (0.697,1.507) | (0.497,2.608) | (0.666,1.399) | (0.659,3.227) | (0.467,2.001) | (0.923,2.535) | (0.421,1.383) |
| Currently married/partnered | 0.862 | 1.671 | 0.610^ | 1.012 | 0.756 | 1.111 | 0.749 |
|  | (0.562,1.323) | (0.728,3.834) | (0.359,1.038) | (0.401,2.555) | (0.350,1.632) | (0.593,2.081) | (0.405,1.385) |
| **Current school status** | |  |  |  |  |  |  |
| Not in school (ref) | |  |  |  |  |  |  |
| In school | 1.053 | 0.801 | 0.794 | 1.21 | 0.924 | 1.274 | 1.35 |
|  | (0.686,1.615) | (0.314,2.042) | (0.408,1.544) | (0.393,3.730) | (0.375,2.273) | (0.614,2.647) | (0.569,3.203) |
| **Children in home (#)** | 0.962 | 0.982 | 1.05 | 1.006 | 1.068 | 1.018 | 0.978 |
|  | (0.871,1.064) | (0.861,1.121) | (0.976,1.130) | (0.904,1.119) | (0.984,1.158) | (0.939,1.103) | (0.911,1.051) |
| **Rurality** |  |  |  |  |  |  |  |
| Urban home (ref) | |  |  |  |  |  |  |
| Rural home | 1.327 | 1.308 | 0.956 | 0.929 | 1.136 | 1.835* | 1.063 |
|  | (0.914,1.927) | (0.680,2.518) | (0.591,1.544) | (0.546,1.579) | (0.682,1.891) | (1.044,3.226) | (0.578,1.957) |
| **Employment in past year** | |  |  |  |  |  |  |
| Not employed (ref) | |  |  |  |  |  |  |
| Employed | 0.852 | 0.927 | 0.822 | 1.542^ | 1.394^ | 0.777 | 1.087 |
|  | (0.629,1.156) | (0.535,1.605) | (0.565,1.197) | (0.928,2.563) | (0.944,2.058) | (0.527,1.147) | (0.807,1.464) |
| **Wealth quintile** | |  |  |  |  |  |  |
| Lowest wealth (ref) | |  |  |  |  |  |  |
| Low wealth | 1.214 | 1.008 | 0.98 | 1.789 | 1.24 | 1.443 | 1.229 |
|  | (0.785,1.877) | (0.395,2.574) | (0.582,1.651) | (0.669,4.783) | (0.704,2.182) | (0.695,2.995) | (0.837,1.804) |
| Medium wealth | 1.24 | 0.797 | 1.046 | 0.993 | 2.168** | 3.425** | 0.971 |
|  | (0.760,2.023) | (0.325,1.955) | (0.559,1.958) | (0.407,2.423) | (1.263,3.723) | (1.616,7.256) | (0.654,1.442) |
| High wealth | 1.393 | 0.784 | 1.887 | 1.199 | 1.695* | 2.730* | 1.041 |
|  | (0.851,2.281) | (0.302,2.034) | (0.819,4.346) | (0.487,2.951) | (1.024,2.806) | (1.233,6.043) | (0.574,1.888) |
| Highest wealth | 1.381 | 1.027 | 0.430^ | 0.868 | 2.105* | 3.547** | 1.252 |
|  | (0.754,2.528) | (0.364,2.897) | (0.175,1.056) | (0.293,2.573) | (1.056,4.195) | (1.538,8.181) | (0.646,2.425) |
| N | 2454 | 1584 | 2070 | 1007 | 1195 | 1433 | 2455 |
| 95% confidence intervals in brackets | | | | | | |  |
| ^ p < 0.10, * p < 0.05, ** p < 0.01, *** p < 0.001 | | | | |  |  |  |
